# Reusable respirators as personal protective equipment in clinical practice: User experience in times of a pandemic

**DOI:** 10.1007/s00508-022-02022-1

**Published:** 2022-04-12

**Authors:** Mathias Maleczek, Frédéric Toemboel, Maximiliaan Van Erp, Florian Thalhammer, Bernhard Rössler

**Affiliations:** 1 Department of Anaesthesia, Intensive Care Medicine and Pain Medicine, Medical University of Vienna, Waehringer Guertel 18–20, 1090 Vienna, Austria; 2 Academic Simulation Centre of Vienna, Vienna, Austria; 3 Ludwig Boltzmann Institute for Digital Health and Patient Safety, Vienna, Austria; 4 Department of Urology, Medical University of Vienna, Waehringer Guertel 18–20, 1090 Vienna, Austria; 5 Department of Anesthesia and Intensive Care Medicine, University Hospital Krems, Karl Landsteiner University of Health Sciences, Krems, Austria

**Keywords:** SARS-CoV‑2, COVID, FFP3, Mask, Intensive care

## Abstract

**Background:**

The novel strain of severe acute respiratory syndrome coronavirus 2 is highly contagious; therefore, special emphasis must be given to personal protective equipment for healthcare workers. Reusable elastomeric respirators were previously used in intensive care units (ICU). These respirators include full or half masks and devices modified to accommodate a filter. Although the general comfort of masks used in the ICU has been studied, data comparing multiple types of masks during a pandemic are missing.

**Methods:**

A prospective randomized trial was conducted in an ICU. After standardized training, participants were randomized to use one of three mask types (full, half or snorkelling mask), each fitted with a filter equivalent to a class 3 particle-filtering half mask (FFP3) during one shift. The main outcomes were characteristics of using the mask itself (donning/doffing, quality of seal, cleaning), working conditions with the mask (vision, comfort, perceived safety, communication) and a subjective comparison to single-use FFP2/3 masks.

**Results:**

A total of 30 participants were included in the trial, randomized to 10 participants per group. The masks were worn 6.4 (4.5) times (mean SD) for a total duration of 132 (66) min per shift. The tested masks were rated 7 (2.6) (mean SD) in comparison to FFP2/3 on a Likert scale (0: worst, 10: best). Significant differences between the masks were found in respect to comfort (7/4/8), donning (8/7/9), overall rating (8/5/8) and comparison to single-use FFP2/3 masks (9/7/9; full-, half, snorkelling mask).

**Conclusion:**

Using reusable elastomeric masks is feasible in clinical practice. Full face masks were significantly better in terms of comfort, donning, overall rating and in comparison to single-use FFP2/3 masks.

## Introduction

The novel strain of coronavirus SARS-CoV-2 is highly contagious; therefore, special emphasis has been placed on personal protective equipment (PPE) for healthcare workers (HCW) since the beginning of the outbreak at the end of 2019 [1, 2]. Although studies first reported that nearly 20% of HCWs were infected with SARS-CoV‑2, later studies reported that less than 8% of HCWs were infected, which is still significantly higher than the rate of infection in the general population [2, 3]. This shows the importance of PPE in clinical settings. Unfortunately, PPE use is often challenging or simply unsuitable for the situation. Therefore, a large number of these infections were potentially preventable if PPE had been used correctly [4].

The World Health Organization, European Centers for Disease Control and the US Centers for Disease Control, recommend that HCWs use respirator masks, especially during aerosol-generating procedures such as intubation or bronchoscopy [5–7].

The recommended respirators are N95 (95% particle filtration), FFP (filtering facepiece) 2 (92% particle filtration) or FFP3 (98% particle filtration) [8, 9]. The sudden increase in demand for N95, FFP2/3 masks caused a worldwide shortage of these respirators during the first month of the pandemic in 2020 [10].

Although reusable elastomeric respirators were previously used in clinical settings, single-use masks are currently the most commonly used masks [11–13]. Reusable elastomeric masks are available as half-masks that cover only the nose and mouth, whereas full face masks cover the entire face, including the eyes and cheeks. It was previously reported that reusable elastomeric masks are equally protective when using suitable filters. Although the level of user comfort was lower, HCWs felt more secure when wearing elastomeric half face masks than when wearing N95 masks [11, 14–16]. Reusable masks can be cleaned after use, and filters are designed for prolonged durability and use over several weeks depending on the dust exposure and manufacturer. These advantages led to the use of reusable full face masks, half face masks or modified snorkelling masks during the coronavirus disease 2019 (COVID-19) pandemic [17, 18].

The most important factor regarding masks is safety when used as PPE. Furthermore, work performance decreases due to impaired vision, hearing and speech when using PPE, and the increased work required to breathe and user comfort are also significant concerns [19]. Although user comfort when wearing a mask was studied prior to using elastomeric half face masks, data from a prospective randomized trial comparing multiple types of masks during an active pandemic are missing.

## Patients, material and methods

A prospective randomized trial was conducted at one of the Medical University of Vienna’s intensive care units that treats infectious and postinfectious patients with COVID-19. The local ethics committee waived the need for a statement. The local workers committee and the data safety committee agreed to this study being conducted. Healthcare workers were informed about the trial and provided oral and written consent. The CONSORT guidelines were followed [20]. All procedures followed were in accordance with the ethical standards of the responsible committee on human experimentation (institutional and national) and with the Helsinki Declaration of 1975, as revised in 2008.

The study population consisted of all doctors and nurses working in this particular intensive care unit during the recruitment interval from 29 September 2020 to 8 November 2020. They all provided routine care to patients with COVID-19 and were therefore experienced in using PPE. The study included all HCWs who provided informed consent. Recruitment for the study was performed via email, phone, and personal recruiting by the study team. All HCWs with known pregnancy, history of pulmonary disease, need for optical glasses, and history of stenocardia or shortness of breath during exercise were excluded.

As this was a hypothesis-generating trial, no formal sample size calculation was performed, and 30 HCWs were included.

At the intensive care unit in question, three types of reusable respirators were available apart from FFP2/3 masks: EKASTU Fullface-Mask C607 (EKASTU Safety GmbH, Waiblingen, Germany), OCEAN REEF ARIA UNO + APA-Adapter (Mestel Safety Srl, Milano, Italy) and DRAEGER Half-Masks X‑plore 4740 (Draegerwerk AG & Co. KGaA, Luebeck, Germany). The EKASTU safety filter DIRIN 230 P3R D (EKASTU Safety GmbH) was used. All the equipment was certified as PPE category III following EU regulation 2016/425.

Outcomes of interest were use of the mask itself (donning/doffing, quality of seal, cleaning), working conditions with the mask (vision, comfort, perceived safety, communication) and a subjective comparison to single-use FFP2/3 masks. The survey was conducted over a 6-week period between 2020-09-29 and 2020-11-08.

Participants were asked to rate and record their experiences in a data form and complete a questionnaire regarding the utilization of the mask after randomization. The abovementioned subjects were examined using a 10-point Likert scale, and baseline demographics and timing of PPE use as well as unplanned removal of mask were documented.

At the beginning of the shift, participants were randomized to one of the three models via a digital randomization tool (http://www.randomization.com). A block randomization with a size 6 block was used.

After randomization, the participants were instructed on how to use PPE according to the University’s standard operations procedure. The training included safety precautions, proper use, safe donning and doffing, cleaning, inspecting for damages, precautions in case of emergency and inspecting before use. As blinding of neither the participant nor the study team was feasible, this was an open-label trial.

Masks were marked with the participant’s pseudonym, and participating HCWs were asked to use the mask during one shift when in the patients’ room. To ensure maximum safety for the participants, trial masks were used only when treating postinfectious patients.

Completed questionnaires were digitalized and analyzed using Python 3.8 [21] (primarily pandas and numpy [22]). Descriptive analysis was performed, and χ^2^-tests, ANOVA, Mann-Whitney‑U and Kruskal-Wallis tests were used as appropriate. Likert scale variables were seen as ordinally scaled. *P* < 0.05 was chosen as level of significance.

## Results

During recruitment, 30 HCWs were included in the trial and none were excluded. The median age group of HCWs was 36–40 years of age with a median of 4.5 years of experience. Almost all participants (97%) had prior experience with reusable masks. Each group consisted of 10 HCWs, with no obvious differences between groups regarding demographic details. In total 10 physicians and 20 nurses participated in the trial. The baseline demographics of the included participants are shown in Table 1.

**Table 1:** Demographic details of participants are shown

Mask name		EKASTU		OCEAN REEF		DRAEGER		
		*n*	%	*n*	%	*n*	%	*p*-value
Participants	–	10	33	10	33	10	33	–
Sex	Female	5	50	6	60	5	50	0.999
Age (years)	–	–	–	–	–	–	–	0.928
	21–25	1	10	2	20	3	30	–
	26–30	2	20	3	30	2	20	–
	31–35	2	20	1	10	3	30	–
	36–40	2	20	2	20	1	10	–
	41–45	2	20	1	10	0	0	–
	46–50	0	0	1	10	1	10	–
	51–55	1	10	0	0	0	0	–
Profession	–	–	–	–	–	–	–	0.499
	Nurse	7	70	5	50	8	80	–
	Doctor	3	30	5	50	2	20	–
Work experience (Years) (mean/SD)		11.32/10.22		8.23/9.48		6.2/8.01		0.487
Experience with reusable masks		10	100	10	100	9	90	0.999

*n* number, *SD* 95% standard deviation

The masks were worn 6.4 times (mean) for a total duration of 132 min per shift (mean). Unintended doffing occurred two times: once in the EKASTU group and once in the Draeger group. The Draeger mask was doffed because of neck band dislocation, and the EKASTU mask had to be doffed because of an air leak from the mouthpiece into the face piece.

**Fig. 1:**
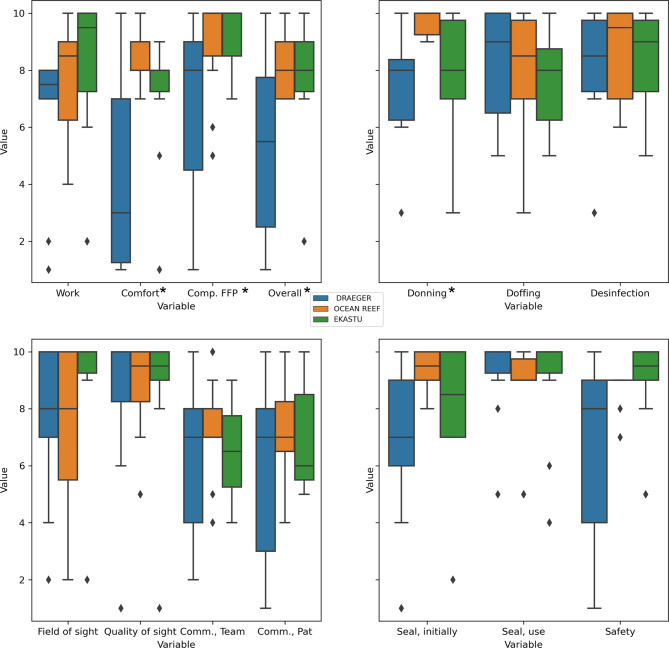
Boxplots of all reported variables regarding mask performance are shown. Value is the value on the Likert scale, *asterisk* indicates reaching a significance level < 0.05. *Comp. FFP* comparison with FFP, *Comm., Team* communication with team, *Comm., Pat.* communication with patients

Using Kruskal-Wallis, significant differences between the groups were found in respect to comfort, donning, overall rating and comparison to single-use FFP2/3 masks. The overall mean rating of the masks was 7 (Likert-like scale: 0: worst, 10: best), with a significant difference between the groups (Kruskal-Wallis, *p* = 0.048). The EKASTU mask mean rating was 7.8 (SD: 2.3), the OCEAN REEF mask rating was 8.1 (SD: 1.1) and the Draeger half-mask rating was 5.2 (SD: 3.2). In comparison to FFP2/3, there was a significant difference as well: EKASTU and OCEAN REEF were rated similarly (9.2 and 8.9), and Draeger was rated 5.2. The overall comfort of the masks was rated 8.2 (mean), with the EKASTU and the OCEAN REEF masks performing best (9.2, 8.9). The Draeger mask was rated 6.6 compared to the single-use FFP2/3 mask. Donning was rated easiest with the ocean reef mask (9.7 vs. 7.7/7.4; EKASTU/Draeger), *p* = 0.004. Details can be found in Table 2. Boxplots of the variables are shown in Fig. 1.

**Table 2:** Overall ratings and ratings per group of the examined outcomes

	All	EKASTU	DRAEGER	OCEAN REEF	*p*-value
*n*	30	10	10	10	–
**Uses, mean (std)**					
Number	6.43 (4.53)	7.10 (5.00)	6.40 (5.80)	5.80 (2.57)	0.82
Time (min)	131.73 (66.08)	142.80 (86.65)	132.70 (66.69)	119.70 (42.61)	0.75
*No unintended doffing (in %)*	92.31	88.89	88.89	100.00	–
*Donning, mean (std)*	8.25 (2.01)	7.70 (2.31)	7.35 (1.97)	9.70 (0.48)	**0.004**
**Mask seal, mean (std)**					
Initially	8.10 (2.41)	8.00 (2.58)	6.90 (2.85)	9.40 (0.70)	0.07
During use	9.00 (1.70)	8.90 (2.13)	9.20 (1.62)	8.90 (1.45)	0.35
*Doffing, mean (std)*	7.80 (2.07)	7.50 (1.72)	8.20 (2.10)	7.70 (2.50)	0.56
*Disinfection, mean (std)*	8.17 (2.09)	8.20 (1.93)	7.70 (2.67)	8.60 (1.65)	0.76
*Perceived safety, mean(std)*	8.13 (2.36)	9.00 (1.56)	6.70 (3.40)	8.70 (0.67)	0.08
*Work, mean (std)*	7.40 (2.47)	8.20 (2.62)	6.40 (2.63)	7.60 (2.01)	0.11
*Field of sight, mean (std)*	7.97 (2.80)	9.00 (2.49)	7.60 (2.80)	7.30 (3.06)	0.25
*Quality of sight, mean (std)*	8.60 (2.43)	8.60 (2.76)	8.40 (2.91)	8.80 (1.69)	0.97
*Comfort, mean (std)*	6.60 (2.95)	7.10 (2.42)	4.40 (3.53)	8.30 (0.95)	**0.04**
**Communication, mean (std)**					
With team	6.60 (2.04)	6.50 (1.58)	6.10 (2.69)	7.20 (1.75)	0.63
With patient	6.64 (2.52)	7.00 (2.16)	5.71 (3.40)	7.12 (1.96)	0.77
*Compared to FFP2/3*	8.23 (2.50)	9.20 (1.32)	6.60 (3.20)	8.90 (1.91)	**0.03**
*Overall rating, mean (std)*	7.03 (2.62)	7.80 (2.30)	5.20 (3.16)	8.10 (1.10)	**0.048**

ANOVA and Kruskal Wallis (for Likert scale) was used as appropriate to compare the groups

*std* standard deviation

Exploratory analysis of the influence of healthcare professionals showed no significant differences between physicians and nurses except for the number of PPE uses during their shift (8.8 vs. 5.25, *p* = 0.04, t test) and the initial seal with a doctors mean rating of 9.1 vs. nurses rating 7.6 (Mann-Whitney U, *p* = 0.03).

## Discussion

Masks are an integral component of protective equipment during a pandemic in which the disease is aerosol-transmitted, such as the current COVID-19 outbreak. Due to the supply chain during pandemic waves, a broad variety of different types of masks, including reusable elastomeric masks, were used. Those reusable masks are an essential alternative to the commonly used single-use masks, but data about their use and safety are sparse. This is the first comparative trial that compares HCW experiences using different elastomeric reusable masks during the COVID-19 pandemic in an ICU while performing daily routine work.

Regarding overall rating, comfort and donning, the snorkelling mask was rated best, with a significant difference compared to the two other groups. Snorkelling masks were suggested for use as PPE during the supply chain shortage in the course of the first wave of the pandemic [23, 24]. Several studies compared these masks to single-use N95 respirators and found comparable data to this trial: Good overall comfort, vision, perceived safety and communication were the greatest challenges [23, 25, 26].

The seal quality did not differ significantly between groups, neither regarding initial seal nor during use. It was rated at 8.1 overall for the initial seal and 9.0 during use. This finding is reassuring, as seal quality is the most important factor regarding employee safety in an airborne pandemic. This finding corresponds to the literature that reported data about mask seal quality in reusable elastomeric masks [27, 28].

Donning and especially doffing are most important in terms of the possibility of contamination: easy donning makes the equipment practical to use and possibly increases PPE use, whereas easy doffing is the leading factor for HCW contamination [29–31]. This makes both donning and doffing essential for workplace safety and therefore of great interest [29, 30, 32–34]. Interestingly, donning was rated easiest in the snorkelling mask, whereas doffing was easiest with the Draeger half mask, although differences were only significant regarding the donning process. This may be due to the well-known mechanism of donning snorkelling masks compared to professional masks. Doffing was rated the same in all three masks with nonsignificant differences.

An important finding of this trial is the risk for unintended doffing that renders the user unprotected against airborne pathogens. This occurred two times: once with a full mask and once with a half mask. This possibly dangerous finding must be further evaluated in future research although one has to keep in mind that this finding is hypothesis generating only.

Working conditions, comfort, communication and field of vision were investigated. Only regarding comfort was a significant difference between the masks found, with the scuba mask performing best, at an absolute difference of nearly four points. A possible confounder regarding the lower comfort ratings of the half mask can be the mandatory use of masks, goggles and face shields together when using half-masks. In contrast to full masks, the use of three devices instead of one can change comfort ratings. This fits into the current literature reporting the use of elastomeric masks [11, 16, 27].

Compared to the single-use FFP2/3 mask, a significant and relevant difference by three points between the devices was found, with an overall rating of 5.2 vs. 7.8 vs. 8.2 (Draeger/EKASTU/OCEAN REEF). The overall comparison of elastomeric masks with FFP2/3 was very good (7 points mean), with significant differences between the groups. Therefore, in our data, reusable elastomeric masks can be seen as superior to single-use masks. Hines et al. report the results of a survey of over 1000 participants after implementing a reusable mask program after the 2009 H1N1 pandemic and subsequent supply shortages. Similar to our results, HCWs felt safer using elastomers in their trial. In contrast, N95 masks were rated more comfortable and easier to communicate [11, 16].

Although our results fit well into the known data, some limitations must be acknowledged. As this was a pilot trial, there were only a small number of participants in a single ICU. Therefore, the results have to be seen not as confirmatory but for hypothesis generation only. All participants had previously used full face masks, resulting in a bias in favor of EKASTU, as they were used similar to elastomeric full face masks, although unfamiliar to the special type used in this trial. Since the half mask had to be worn with goggles and a face shield according to local hospital standards and international recommendations, the additional item has added complexity in donning/doffing and has influenced comfort, visibility, perceived safety, etc. Another limitation is how the comparison with FFP2/3 was done. Due to the broad variety of mask fittings, forms and materials as well as manufacturers, it is not reasonable to use a single FFP2/3 mask type for comparison; instead, the variable comparison to FFP2/3 was introduced to minimize this limitation.

In the future, larger trials—potentially with more mask types—will be needed to determine the perfect mask for use during pandemics. In this trial, we used a variety of masks to cover all available mask types (full face, half face). A direct comparison to FFP2/3 masks will be subject to further research as well. A topic of special interest will be the interperson variability of mask assessment, as this was not conducted in this trial.

This trial will help improve the understanding of the most valuable article of personal protection equipment during this pandemic. The use of reusable elastomeric respirators was perceived to be safe. Full masks were rated better in most of the categories.

## Conclusion

Reusable elastomeric masks are feasible as PPE in an intensive care unit setting where patients with COVID-19 are treated. There were significant differences between the mask types regarding comfort, donning, overall rating and comparison to single-use FFP2/3 masks. Perceived safety was highest in the EKASTU full-face mask, although two incidences of unintended doffing occurred, one with a full mask and one with a half mask.
